# CRISPR screening identifies BET and mTOR inhibitor synergy in cholangiocarcinoma through serine glycine one carbon

**DOI:** 10.1172/jci.insight.174220

**Published:** 2024-01-23

**Authors:** Yan Zhu, Dengyong Zhang, Pooja Shukla, Young-Ho Jung, Prit Benny Malgulwar, Sharmeen Chagani, Medina Colic, Sarah Benjamin, John A. Copland, Lin Tan, Philip L. Lorenzi, Milind Javle, Jason T. Huse, Jason Roszik, Traver Hart, Lawrence N. Kwong

**Affiliations:** 1 Department of Translational Molecular Pathology, The University of Texas MD Anderson Cancer Center, Houston, Texas, USA.; 2 Department of general surgery, The First Affiliated Hospital of Bengbu Medical College, Bengbu, Anhui, China.; 3 Department of Bioinformatics and Computational Biology, The University of Texas MD Anderson Cancer Center, Houston, Texas, USA.; 4 Department of Natural Sciences, Rice University, Houston, Texas, USA.; 5 Department of Cancer Biology, Mayo Clinic Jacksonville, Florida, USA.; 6 Metabolomics Core Facility, Department of Bioinformatics & Computational Biology,; 7 Department of Gastrointestinal Medical Oncology,; 8 Department of Melanoma Medical Oncology-Research, Division of Cancer Medicine,; 9 Department of Genomic Medicine, Division of Cancer Medicine, and; 10 Department of Cancer Biology, The University of Texas MD Anderson Cancer Center, Houston, Texas, USA.

**Keywords:** Cell Biology, Gastroenterology, Drug screens, Liver cancer

## Abstract

Patients with cholangiocarcinoma have poor clinical outcomes due to late diagnoses, poor prognoses, and limited treatment strategies. To identify drug combinations for this disease, we have conducted a genome-wide CRISPR screen anchored on the bromodomain and extraterminal domain (BET) PROTAC degrader ARV825, from which we identified anticancer synergy when combined with genetic ablation of members of the mTOR pathway. This combination effect was validated using multiple pharmacological BET and mTOR inhibitors, accompanied by increased levels of apoptosis and cell cycle arrest. In a xenograft model, combined BET degradation and mTOR inhibition induced tumor regression. Mechanistically, the 2 inhibitor classes converged on H3K27ac-marked epigenetic suppression of the serine glycine one carbon (SGOC) metabolism pathway, including the key enzymes PHGDH and PSAT1. Knockdown of PSAT1 was sufficient to replicate synergy with single-agent inhibition of either BET or mTOR. Our results tie together epigenetic regulation, metabolism, and apoptosis induction as key therapeutic targets for further exploration in this underserved disease.

## Abstract 2

<p>CRISPR screening identifies synergy between BET and mTOR inhibition&nbsp;against cholangiocarcinoma through serine glycine one carbon metabolism.</p>

## Graphical abstract

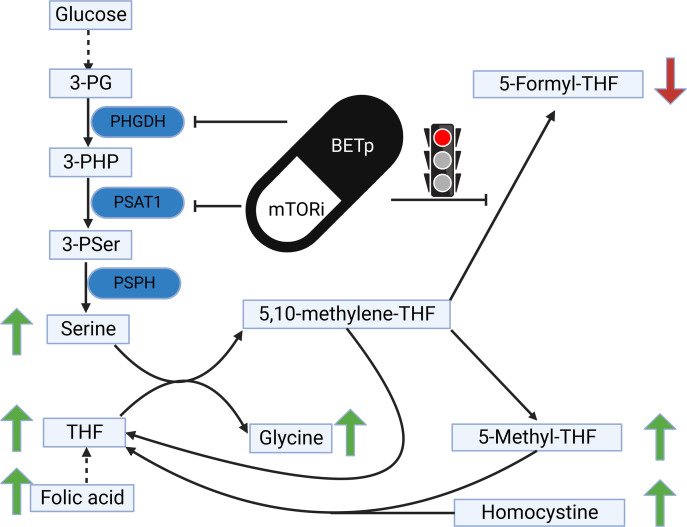

## Introduction

Cholangiocarcinoma (CCA) is a malignancy of the biliary tract epithelium and is the second most common primary liver tumor following hepatocellular carcinoma (HCC); it accounts for ~15% of primary liver cancers and ~3% of gastrointestinal malignancies (1, 2). Due to late diagnoses, poor prognoses, and limited treatment strategies, CCA shows a dismal outcome with a less than 5% overall 5-year survival and only a median overall survival of ~7 months (3). Moreover, the incidence (0.3–6 per 100,000) and mortality (1–6 per 100,000) of CCA is increasing globally over recent decades (4–6). For early CCA, surgical resection or liver transplantation with chemotherapy or radiotherapy can be potentially curative, but postoperative complications are severe and fewer than 10% of patients are eligible (7, 8). For patients with advanced or unresectable CCA, chemotherapeutic options are few, with gemcitabine and cisplatin as the standard treatment showing limited effectiveness (9). Intraarterial therapy and immune checkpoint therapies also show limited effectiveness against CCA (10, 11).

Next-generation sequencing studies have revealed that CCA is a highly epigenetically dysregulated disease, and this epigenetic characteristic provides new strategies for drug treatment. Mutations in several epigenetic genes with high frequency have been reported in CCA, including the oncogene *IDH1/2* (1%–15%) and the tumor suppressor genes *BAP1* (1%–23%), *ARID1A* (2%–11%), *ARID2* (1%–5%), and *PBRM1* (1%–23%) (12), as well as general epigenetic dysregulation regardless of the mutation status of these genes, such as widespread DNA hypermethylation (13). Such dysregulation may provide a clue for precision medicine. For example, CCA cell lines harboring an *IDH1* mutation are more sensitive to inhibition of Bromodomain and Extra-Terminal (BET), a family of epigenetic readers and transcriptional coactivators that combine acetylation of histone tails and recruitment of positive transcription elongation factor b (P-TEFb) (14, 15). Moreover, loss-of-function mutations in *ARID1A* in breast cancer lead to hyperacetylation of histone H4 and facilitation of BET-induced transcription, also leading to sensitivity to small molecule BET inhibitors (BETi) (16). This sensitivity was also seen in ARID1A-mutant ovarian cancer cells, and that may in part be due to BETi downregulating ARID1B, a known synthetic lethal event for cells harboring ARID1A loss (17). In CCA, BETi efficacy may not be limited to only IDH1 and ARID1A mutations. Thus, we considered BET inhibition as a starting point for identifying novel combination therapies for CCA.

ARV825 is a BET PROTAC (hereafter referred to as “BETp”) that contains a triazolo-diazepine acetamide Brd4-binding moiety, a cereblon binding moiety, and a polyethyleneglycol linker (18). ARV825 recruits Brd4 — and, to a lesser extent, Brd3 and Brd2 — to the E3 ubiquitin ligase cereblon to promote their degradation by ubiquitination and proteasome-mediated degradation. ARV825 or its close sister compound ARV771 have shown efficacy alone or in drug combinations in multiple mouse models including AML (19, 20), DLBCL (21), mantle cell lymphoma (22), and prostate cancer (23). ARV825 also showed an increased induction of cell cycle arrest and apoptosis in CCA cell lines compared with the traditional Brd4 inhibitors JQ1 or OTX015 (24).

Here, we conducted a genome-wide CRISPR screen and identified a synergistic combination effect between BETp and ablation of members of the mammalian target of rapamycin (mTOR) pathway. The efficacy of combined inhibition of BET and mTOR was then validated through in vitro and in vivo experiments utilizing small-molecule mTOR inhibitors, while mechanistic analyses identified a synergistic decrease in global H3K27ac at enhancers as underlying transcriptional suppression of serine glycine one carbon (SGOC) metabolism. This research provides a proof of principle for exploring the combination of BET + mTOR inhibition in the treatment of CCA.

## Results

### CCA cell lines are sensitive to BETp ARV825.

**Figure 1:**
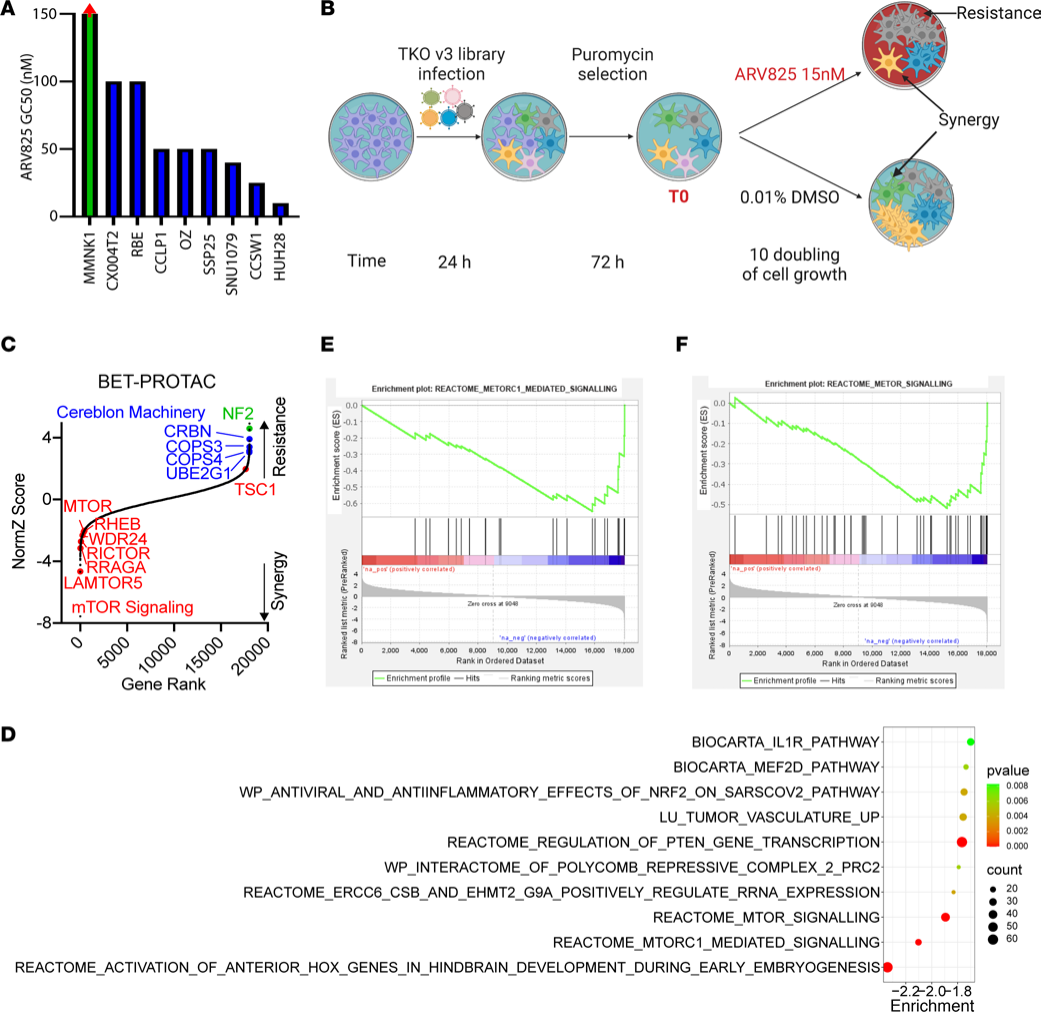
In vitro CRISPR screen identified synergy with BET inhibition. (**A**) BETp ARV825 GC50 for normal cholangiocyte (MMNK1) and 8 CCA cell lines. GC50 for MMNK1 is > 150 nM. (**B**) Schematic of the workflow for CRISPR screening with ARV825. (**C**) DrugZ ranking of CRISPR screen hits in SNU1079 with ARV825. Data are from 2 independent biological repeats. (**D**) Gene set enrichment analysis of depleted sgRNAs. (**E** and **F**) The top mTOR-related gene sets from GSEA.

To identify any potential correlation between BETp sensitivity and CCA mutations, we first sequenced 300+ cancer-related genes in 8 CCA cell lines through a targeted NGS panel. As shown in Supplemental Table 2 (supplemental material available online with this article; https://doi.org/10.1172/jci.insight.174220DS1), *IDH1* mutations were found in SNU1079 (*IDH1*^R132C^) and RBE (*IDH1*^R132S^); *ARID1A* mutations were found in SNU1079 (*ARID1A*^L2029fs^), HUH28 (*ARID1A*^M759V^), and CX004T2 (*ARID1A*^K1928R^ and *ARID1A*^E1935D^); an *ARID2* mutation was found in HUH28 (*ARID2*^Q1575X^); and a *BAP1* mutation of unknown importance was found in CCLP1 (*BAP1*^C649Y^). Six of these lines (excluding CX004T2 and OZ) have publicly available NGS data, with which our results agreed. We then tested the BETp ARV825 in these 8 CCA cell lines as well as the normal immortalized cholangiocyte cell line MMNK1. As shown in Figure 1A, most of the CCA cell lines showed growth inhibitory half-maximal concentration (GC50) ranging from 10 to 100 nM, while the GC50 for MMNK1 was over 150 nM, suggesting a degree of sensitivity and specificity of ARV825 against CCA over normal cells. There was no clear correlation between BETp sensitivity and any gene or gene family mutation status. Since SNU1079 showed moderate growth rate and sensitivity to ARV825, it was selected for CRISPR screening to identify both BETp-sensitizing and resistance-conferring sgRNAs.

### A whole-genome dropout CRISPR screen suggested a synergistic effect between mTOR complex impairment and BETp.

We conducted a whole-genome CRISPR screen with or without BETp in SNU1079 (Figure 1B). To validate the reliability of our results, we first analyzed the screen results without BETp. We found that 509/515 (99%) of known essential genes (25) were depleted, while sgRNAs targeting bona fide tumor suppressor genes were strongly enriched, including *TP53*, *TP53BP1*, *CDKN1A*, and *KEAP1* (Supplemental Table 3). These results together support the reliability of the screen.

Next, using the DrugZ algorithm, we identified sgRNAs that were significantly changed in the presence of ARV825 treatment as compared with control (26). The full data are provided in Supplemental Table 4. We first examined enriched sgRNAs, which would indicate that they confer resistance to BETp. As shown in Figure 1C, NF2 (in green) was the second-most enriched gene, consistent with the results of a CRISPR screen in non–small cell lung cancer, in which sgNF2 induced resistance to the BETi JQ1 (27). Additionally, sgRNAs related to the E3 ubiquitin ligase Cereblon (28) were also enriched after drug treatment, as expected, since loss of these genes would attenuate BET ubiquitination induced by ARV825; these genes include *CRBN*, *COPS3*, *COPS4*, and *UBE2G1* (Figure 1C, in blue). These results further underscore the technical and biological fidelity of the results.

Finally, we identified sgRNAs depleted in the presence of ARV825. Gene Set Enrichment Analysis (GSEA) was used to identify enriched pathways ranked by their DrugZ score (Figure 1D). mTOR1-mediated signaling (Figure 1E) and mTOR signaling (Figure 1F) gene sets were among the top 3 hits. These included the bona fide mTOR components/regulator proteins *LAMTOR5*, *RRAGA*, *RICTOR*, *WDR24*, *RHEB*, and *MTOR* itself (Figure 1C, in red) as top individual gene hits. mTOR is the core component of both mTOR complex 1 (mTORC1) and mTORC2, while Rictor is an essential part of mTORC2. Lamtor5 (part of the Ragulator complex), Rraga (part of the Rag complex that binds Ragulator), and Wdr24 (part of the Gator2 complex that activates Rag) work together to activate mTORC1 signaling through sensing amino acid abundance and by allowing mTORC1 to interact with the G protein Rheb (29). Moreover, *TSC1*, a well-established negative regulator of Rheb and mTORC1 signaling, was a resistance-conferring hit. Overall, the results of this CRISPR screen suggested a synergistic effect between BETp and the inhibition of mTORC1 and/or mTORC2.

### BETp or BETi plus mTORi shows synergy against CCA in vitro.

**Figure 2:**
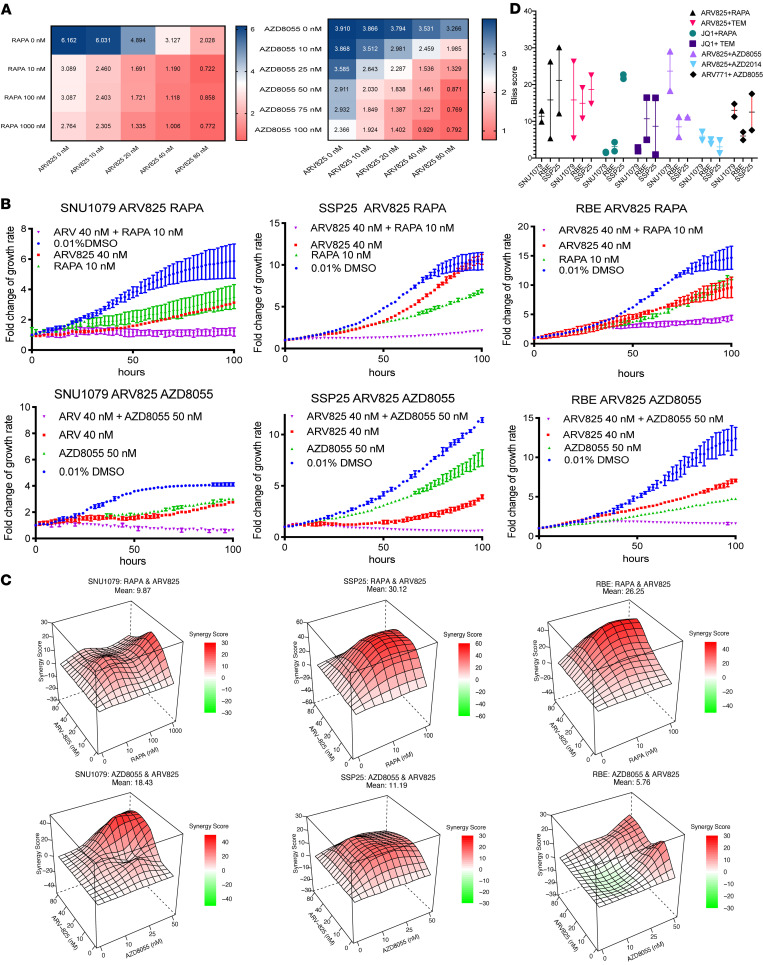
In vitro validation of synergy between mTOR and BET inhibition. (**A**) Growth fold of SNU1079 with 100 hours under the combination of RAPA/AZD8055 and ARV825. (**B**) Growth curve of 3 cell lines under the given BET and mTOR inhibition states. (**C**) Bliss score curves of 3 cell lines under the given BET and mTOR inhibition states. (**D**) Summary of highest Bliss scores in 3 CCA cell lines with different BET and mTOR inhibitor combinations with duplicates, excluding 80 nM ARV825, which may have off-target effects. Data are from 2 independent biological repeats and are presented as mean ± SD.

To validate the predicted synergistic effect between BET and mTOR small molecule inhibition, we first selected the mTORC1 inhibitor (mTORC1i)rapamycin (“RAPA”) and the dual mTORC1/2 inhibitor AZD8055. When combined with ARV825, either RAPA or AZD8055 showed strong synergy in 3 tested CCA cell lines: SNU1079, SSP25, and RBE (Figure 2, A and B), with mean Bliss scores ranging from 10 to 25 at the highest peaks (Figure 2C). As little as 10 nM RAPA was sufficient for strong synergy and 50 nM for AZD8055, while ARV825 typically required at least 40 nM for strong synergy. After 100 hours of treatment, combination-treated cells were sparse and adopted an elongated morphology compared with control or single-treated groups and showed strong growth suppression in all tested cell lines (Supplemental Figure 1, A and B).

To confirm that our results were not limited to the above agents, we also tested additional compounds: the mTORC1i temsirolomus (TEM); the mTORC1/2i AZD2014; the BETp ARV771, a close sister compound to ARV825; and the BETi JQ1 in the following combinations: TEM + ARV825, AZD2014 + ARV825, AZD8055 + ARV771, RAPA + JQ1, and TEM + JQ1. Regardless of the combination tested, synergy was seen as measured by Bliss scores in all 3 cell lines (Figure 2D), accompanied by longitudinal growth suppression (Supplemental Figures 2 and 3). We note that low synergies were seen in combinations containing JQ1, suggesting that BETp may have higher efficacy over BETi. In total, these combination therapy results validated a synergistic effect between BET and mTOR inhibition against CCA in vitro.

### BETp plus mTORi enhances cell cycle arrest and apoptosis compared with single agents.

**Figure 3:**
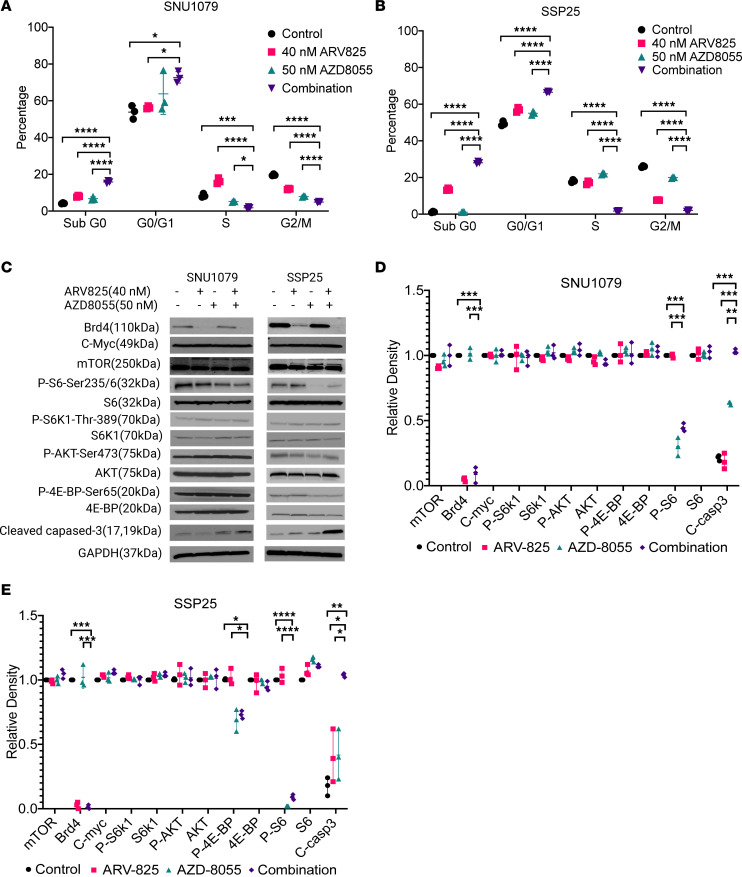
BETp plus mTORi enhances cell cycle arrest and apoptosis compared with single agents. (**A** and **B**) Cell cycle measurement by propidium iodide in SNU1079 (**A**) or SSP25 (**B**) at 48 hours with the listed treatments. Data are from 3 independent biological repeats and are presented as mean ± SD. One-way ANOVA was used to calculate statistical difference. (**C**) Western blots of known target proteins for BET and mTOR inhibition at 48 hours with the listed drug treatments in SNU1079 and SSP25. (**D** and **E**) Quantitative measurement of protein levels from **C**, normalized to GAPDH. Data are from 3 independent biological repeats and presented as mean ± SD. One-way ANOVA was used to calculate statistical difference. ****P < 0.0001; ***P < 0.001; **P < 0.01; *P < 0.05. See full unedited blots in supplemental material.

We next examined the mode of CCA growth suppression using propidium iodide (PI) staining and flow cytometry to evaluate the effects of the BETp + mTORi combination on cell proliferation and apoptosis. First, we noted that the S and G2/M phases were reduced in both cell lines by the combination compared with single-agent treatments while G0/G1 increased, consistent with G0/G1 cell cycle arrest (Figure 3, A and B). Moreover, the sub-G0 phase also showed a significant increase after combination treatment compared with single agents in both cell lines (Figure 3, A and B), suggesting an increase in cell death. Consistent with this being due to an increase in apoptosis, we found that cleaved caspase-3 was increased by the drug combination in both cell lines over either single agent (Figure 3, C–E). These results suggest that BETp + mTORi enhances both cell cycle arrest and apoptosis in CCA.

To confirm the target engagement of both drugs, we conducted Western blotting (Figure 3, C–E) and demonstrated that ARV825 induced the total degradation of Brd4, while AZD8055 downregulated the downstream mTOR marker phospho-S6 (p-S6) in both cell lines, either alone or when in combination. We note, however, that AZD8055, a dual mTORC1/2i, did not affect pS6K1, p4EBP1, or pAKT levels in either cell line, suggesting that it acts independently of these in targeting pS6 in CCA. We also found no change in the amount of c-Myc in response to ARV825 in either cell line, which is typically considered the most common downstream target of BET (24), suggesting that ARV825 might exert anticancer effects through a different mechanism in CCA than in other cancer types.

### BETp + mTORi reduces global H3K27 acetylation and affects SGOC metabolism through PSAT1.

**Figure 4:**
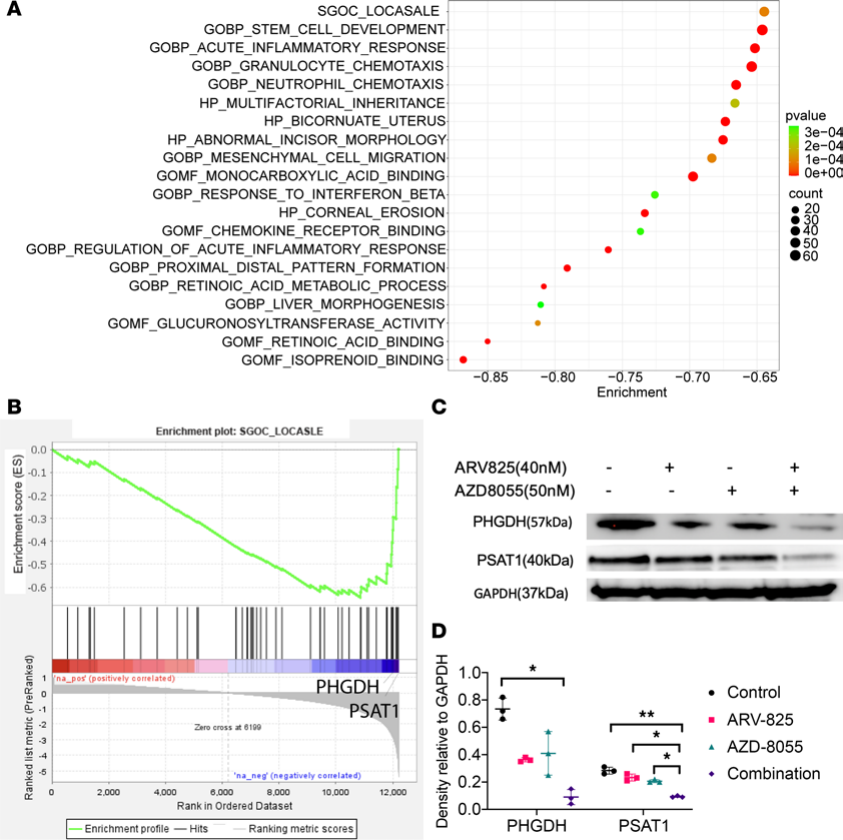
BETp + mTORi affects SGOC metabolism through PSAT1 and PHGDH. (**A**) GSEA of genes with decreased expression under 48 hours of treatment with BETp + mTORi in SNU1079 and SSP25. (**B**) GSEA graph of the SGOC LOCASALE gene set. (**C**) Western blot of PHGDH and PSAT1 at 48 hours with the listed drug treatments in SNU1079. (**D**) Quantitative measurement of **C**, normalized to GAPDH. Data are from 3 independent biological repeats and are presented as mean ± SD. One-way ANOVA was used to calculate statistical difference. **P < 0.01; *P < 0.05. See full unedited blots in supplemental material.

To determine the underlying mechanisms of the BETp + mTORi combination, we conducted RNA-Seq on control, single agents, and the combination in both SNU1079 and SSP25. Based on GSEA results (Figure 4, A and B), SGOC metabolism was a highly enriched gene set. PHGDH and PSAT1, which are 2 main enzymes of the SGOC pathway, were the 2 top enriched genes within the signature and showed a stepwise decrease in expression from control to single and combined agents at both the RNA (Supplemental Figure 4A and Supplemental Table 5) and protein levels (Figure 4, C and D).

**Figure 5:**
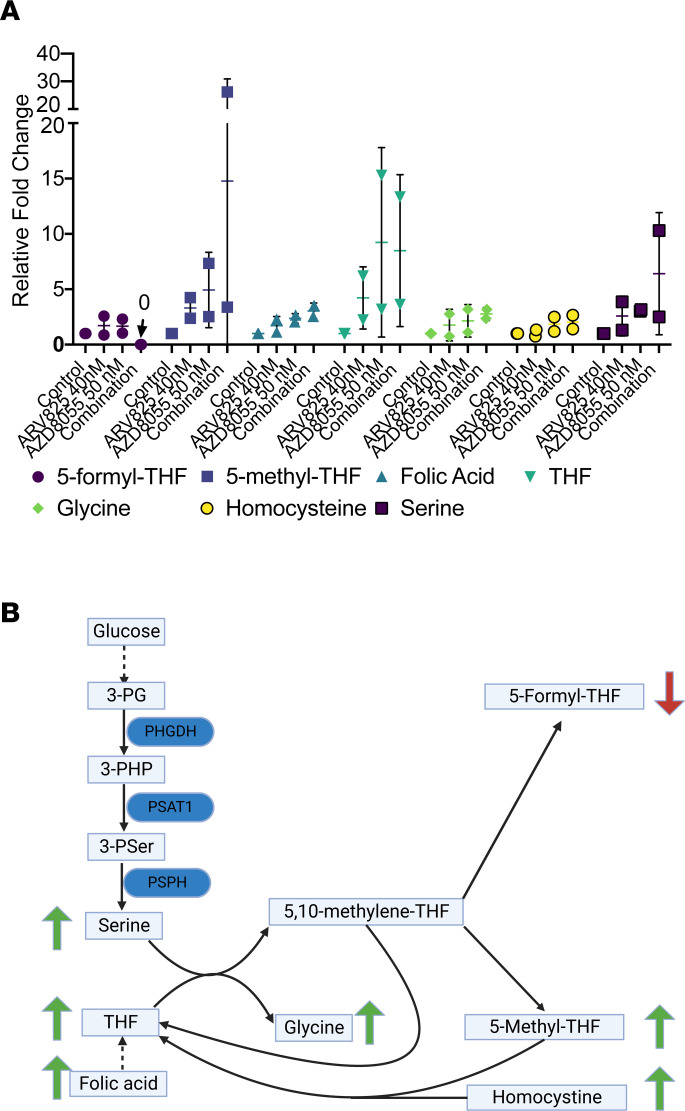
BETp + mTORi affects components of SGOC metabolism. (**A**) MS quantitation of key SGOC components at 48 hours with the listed drug treatments. THF, tetrahydrofolate. (**B**) A simplified schematic of the SGOC pathway with the MS results overlaid. 3-PG, 3-phosphoglycerate; 3-PHP, 3-phosphate hydroxypyruvate; 3-PSer, 3-phosphoserine; PHGDH, phosphoglycerate dehydrogenase; PSAT, phosphoserine aminotransferase; PSPH, phosphoserine phosphatase.

To further validate SGOC dysfunction, we conducted a liquid chromatography–mass spectrometry (LC-MS) analysis of known SGOC components. As shown in Figure 5A, the final product of the pathway, 5-formyl-tetrahydrofolate (5-formyl-THF), was slightly decreased by each single-agent drug but was rendered completely undetectable in all replicates by combined BETp + mTORi. By contrast, all intermediate products increased in the combination group, suggesting that it caused the SGOC process to become “stuck” (Figure 5B).

**Figure 6:**
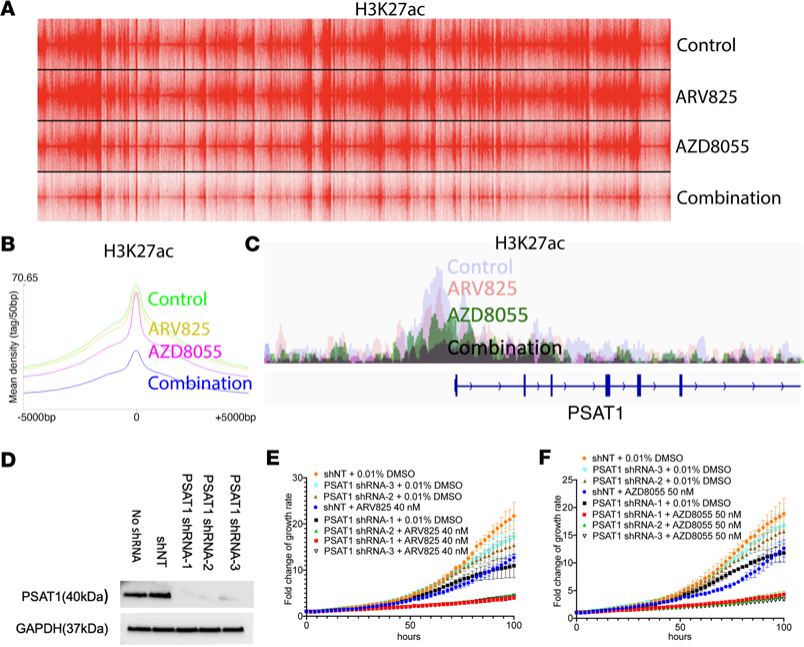
BETp + mTORi reduces global H3K27 acetylation, and PSAT1 knockdown synergizes with BETp and mTORi. (**A**) Global H3K27ac density at 48 hours with the listed drug treatments in SNU1079. (**B**) Summary of global H3K27ac peaks from **A**. (**C**) H3K27ac peaks near the PSAT1 locus in SNU1079. (**D**) Western blot of PSAT1 in shNT or PSAT1 shRNA–transduced SNU1079. (**E** and **F**) Growth curve of SNU1079 with PSAT1 shRNAs combined with 40 nM ARV825 (**E**) or 50 nM AZD8055 (**F**). Data are from 2 independent biological repeats and are presented as mean ± SD. See full unedited blots in supplemental material.

Considering the known epigenetic role of BET at enhancers and super-enhancers (30), we conducted H3K27ac ChIP-Seq in the presence or absence of drug. Interestingly, BETp alone had no effect on H3K27ac levels, whereas mTORi slightly decreased global H3K27ac (Figure 6, A and B); however, the addition of BETp to mTORi drastically decreased the global density of active enhancers, suggesting a synergistic effect. This also held true specifically at the PSAT1 locus (Figure 6C), in which H3K27ac peaks were further decreased by BETp + mTORi over either single agent, suggesting epigenetic regulation as a mechanism of SGOC modulation.

To validate SGOC dysfunction as an underlying mechanism of synergy between BETp and mTORi, we used 3 shRNAs to knock down PSAT1 (Figure 6D). As shown in Figure 6, E and F, PSAT1 knockdown by itself had no effect on cell growth in vitro, yet it significantly increased sensitivity to either ARV825 or AZD8055 compared with nontargeting shRNA, phenocopying the synergy between the 2 inhibitors. Finally, administration of exogenous S-adenosylmethionine (SAM), a key product of SGOC (31), did not rescue the sensitivity to BETp + mTORi in either SNU1079 or SSP25 (Supplemental Figure 4, B and C), suggesting a SAM-independent role of SGOC dysfunction in drug efficacy. Taken together, our results suggest that BETp + mTORi synergistically reduce H3K27ac levels both globally and at PSAT1, resulting in PSAT1 downregulation and SGOC dysfunction as a key mechanism of therapeutic synergy.

### BETp plus mTORi induces CCA regression in vivo.

**Figure 7:**
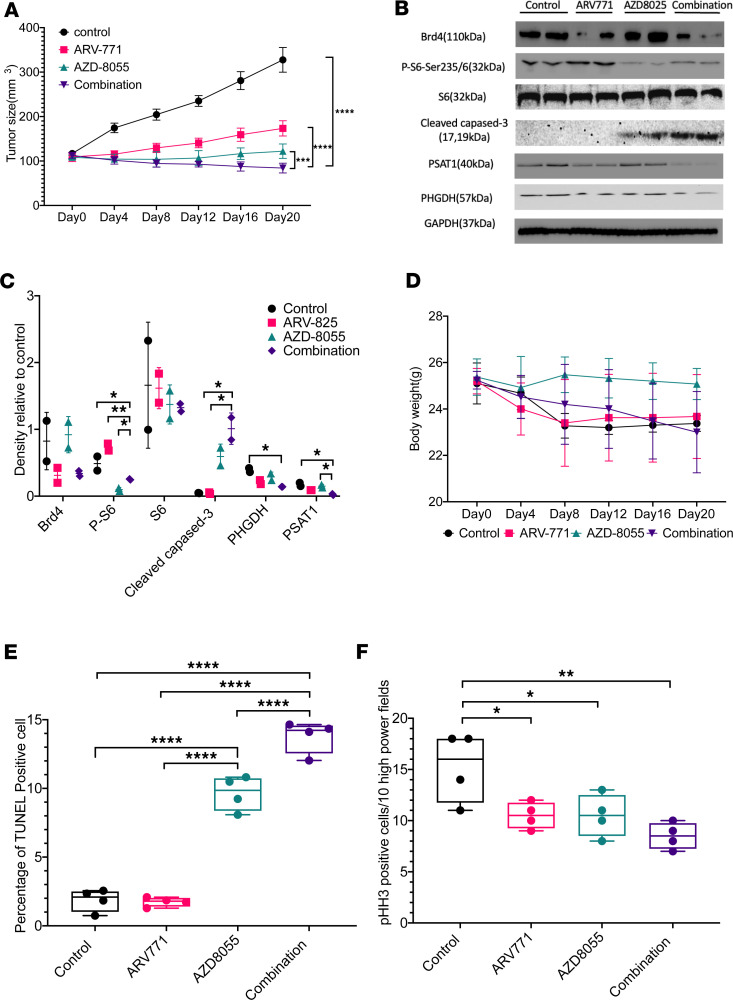
In vivo validation of the synergistic effect. (**A**) Tumor growth curve under different BETp and mTORi treatments. Data are from 8 independent biological repeats and are presented as mean ± SD. One-way ANOVA was used to calculate statistical difference. (**B**) Western blot of selected proteins from the tumors in **A** at day 20. (**C**) Quantitative measurement of protein levels from **B**, normalized to GAPDH. Data are from 2 independent biological repeats and are presented as mean ± SD. One-way ANOVA was used to calculate statistical difference. (**D**) Body weight of the mice in **A**. (**E**) Quantitative measurement of TUNEL assay. Data are from 4 independent tumors and are presented as mean ± SD. One-way ANOVA was used to calculate statistical difference. (**F**) Quantitative measurement of pHH3. Data are from 4 independent tumors and are presented as mean ± SD. One-way ANOVA was used to calculate statistical difference. ****P < 0.0001; ***P < 0.001; **P < 0.01; *P < 0.05. See full unedited blots in supplemental material.

Lastly, we tested the combination in vivo, using the BETp ARV771 instead of ARV825 due to improved bioavailability (32), in the SNU1079 cell line as a s.c. xenograft model. After 20 days of drug administration using a 5-day-on, 2-day-off schedule, the drug combination induced tumor regression, a significant difference when compared with either single agent (Figure 7A and Supplemental Figure 5A). While all 3 drug-treatment groups resulted in a decreased body weight, there was no statistically significant difference between them (Figure 7D). We then assessed mechanism. Consistent with the in vitro results, on-target drug engagement was seen with expected significant decreases in Brd4 and pS6; moreover, we saw a synergistic loss of PSAT1 and PHGDH in the combination group, further supporting the relevance of SGOC (Figure 7, B and C). Additionally, the combination significantly increased the amount of cleaved caspase-3 compared with the single-agent groups. Consistent with this, TUNEL staining confirmed a significant synergistic increase in apoptosis in the combination group compared with the control or single-agent groups (Figure 7E and Supplemental Figure 5B). Next, we found that, although phospho-histone H3 (pHH3, a marker of mitosis) levels were lowest in the combination group, the trend was nonsignificant compared with single agents (Figure 7F and Supplemental Figure 5B). This suggests that, at the 20-day time point, while BETp + mTORi generated tumor regression through the induction of both apoptosis and cell cycle arrest, the synergy was primarily at the level of apoptosis. These results constitute a proof of principle of the efficacy and mechanisms of cotargeting BET and mTOR in CCA.

## Discussion

In this study, we conducted a genome-wide CRISPR screen with BETp in CCA and identified a synergistic combination effect with ablation of the mTOR pathway. The efficacy of combined inhibition of BET and mTOR was then validated through in vitro and in vivo experiments utilizing first- and second-generation mTOR inhibitors and BETi/PROTACs. Moreover, mechanistic analyses using RNA-Seq and ChIP-Seq identified a synergistic decrease in global H3K27ac at enhancers, shown as underlying dysfunction of the SGOC metabolism pathway; the pathway’s suppression is sufficient to replicate synergy with either single-agent BETp or mTORi.

Our identification of SGOC as a potentially novel mechanism of BET + mTOR inhibitor synergy stands in contrast to results from other cancers treated with this combination. For example, in colorectal cancer, the addition of JQ1 to mTOR inhibitors was thought to synergize through suppressing oncogenic kinases (33), while in rhabdomyosarcoma, combined BETi + mTORC1/2i–induced necroptosis-mediated cell death. We also note that a recent study in CCA showed that combination of the dual PI3K/mTOR inhibitor BEZ235 with JQ1 showed efficacy in vitro and in vivo through inhibiting c-Myc and YAP (34). Moreover, in Burkitt lymphoma cells, PSAT1 and PHGDH were regulated by c-Myc and ATF4 (35), while in fibroblasts, mTORC1 can stimulate the expression of PSAT1 and PHGDH through activating ATF4 (36). By contrast, MYC and ATF4 levels were unaffected in our CCA cells by any treatment (Supplemental Figure 4A). Thus, our results add to the understanding of this drug combination and may identify drug and/or cancer type–specific differences in the BETp-mTORi-SGOC mechanism.

We note that BETp are currently not ready for clinical use (37), and furthermore, although there was no significant difference in body weight changes, the combination was on a downward trend (Figure 7D), suggesting room for improving the drug formulation, dosing, and/or schedule. Nevertheless, our results provide a critical proof of concept for dual-pathway targeting and anticipate improvements to both inhibitor classes. Moreover, our mechanistic studies uncovered SGOC as a critical weakness in CCA, suggesting additional ways in which future combined targeting approaches may be explored with greater efficacy and safety profiles.

In summary, an unbiased genome-wide CRISPR screen identified a synergistic combination effect between BETp and impairment of the mTOR pathway, leading to proofs of principle that combined small molecule BETi and mTORi can lead to efficacy against multiple CCA models. Our results tie together epigenetic regulation, metabolism, and apoptosis induction as key therapeutic targets for further exploration in this underserved patient population.

## Methods

### Cell lines and cell culture.

SNU1079 was purchased from the Korean Cell Line Bank, CX004T2 was produced in house, and the other CCA cell lines were purchased from the RIKEN Cell Bank. All cell lines were cultured in high glucose DMEM (Corning, 10-017-CV) containing 10% (v/v) FBS (Thermo Fisher Scientific, 26140079) and 1% (v/v) penicillin/streptomycin (Thermo Fisher Scientific, 15-140-122) in an incubator at 37°C with 5% CO_2_. A list of all inhibitors and antibodies used is provided in Supplemental Table 1.

### T200 sequencing.

For T200 sequencing — a high-depth targeted exome sequencing platform to identify more than 300 of the most common cancer genes **—** DNA was extracted from 8 CCA cell lines by Genomic DNA Purification Kit (Thermo Fisher Scientific, A30701) and quantified using a double-stranded DNA PicoGreen assay, with a quality-control cutoff of > 50 ng/mL, optical density 260/280 between 1.8 and 2.0, and high molecular weight. The T200 next-generation sequencing–targeted sequencing panel was performed at the MD Anderson Sequencing Core. Mutation Annotation Format files were generated through a dedicated pipeline, and all mutations were manually assessed to ensure accuracy.

### CRISPR screening.

To identify genes as synergistic with BETp, SNU1079 was infected with the well-established TKOv3 library containing 71,090 sgRNAs to target 18,053 genes (38). After infection, cells were treated with puromycin to enrich sgRNA-containing cells for 72 hours. At the end of the selection, cells were cultured to reach 10 population doublings in the presence or absence of 10 nM ARV825 before DNA extraction. PCR and next-generation sequencing were used to identify relative enriched (drug resistance–causing) and depleted (drug sensitivity–causing) sgRNAs. The synergy/resistance interactions were analyzed by the DrugZ algorithm (26). GSEA was accomplished through the software GSEA_4.1.0 (39).

### Cell growth curves.

To observe cell growth, 2,000 cells were cultured in each well of a 96-well plate. After the attachment of cells, the appropriate drugs were added to the media to achieve final concentrations. Cell growth rate and images were obtained using the Sartorius Essen IncuCyte ZOOM Live Cell Analyzer. Bliss scores were calculated using the SynergyFinder software (40). A positive value suggests synergy, and a value > 10 suggests strong synergy. One-way ANOVA was used to calculate the significance between different groups.

### Western blot.

Total protein extracts or nuclear protein extracts were prepared from cells or tumors and loaded into each lane of a 4%–12% SDS-PAGE gel. After electrophoresis, total proteins were transferred onto a nitrocellulose membrane. Membranes were blocked in TBS-T buffer (20 mM Tris base, 150 mM NaCl, 0.05% Tween 20, pH 7.4) containing 5% nonfat milk at room temperature for 2 hours prior to the addition of primary antibody and incubation at 4°C overnight. All first antibodies were diluted at 1:1,000 in TBS-T-5% nonfat milk. A list of all antibodies used is provided in Supplemental Table 1. TBS-T–washed membranes were incubated with goat anti-rabbit or goat anti-mouse secondary antibodies in TBS-T–5% nonfat milk (1:10,000) and washed with TBS-T, and membrane-bound antibody was visualized in a chemiluminescence assay. GAPDH was used for normalization.

### Apoptosis analysis by flow cytometry.

PI staining and flow cytometry (BD Accuri C6 Flow Cytometer) were used to evaluate apoptosis (41). In general, 1 × 10^6^ cells were collected and washed with PBS. This mixture was added to the cell-counting slides (Nexcelom, CHT4-SD100-014) to count total cells, and cells were stained with trypan blue. After removing PBS through centrifuge at 400*g* for 5 minutes at room temperature, 1 mL of PI was added to resuspend cells and incubated at 4°C for 1 hour. After that, cells were analyzed using a 488 nm laser line.

### Bulk RNA-Seq.

Prior to RNA or DNA extraction, viable cells were counted by mixing 10 μL of trypan blue (MilliporeSigma, 15250061) with 10 μL of cells. Experiments were carried out only when cell viability was > 90%. Total RNA from each group (vehicle, BETp, mTORi, and BETp + mTORi) of CCA cell lines (SNU1079 and SSP25) with 48 hours of drug treatment was isolated (Thermo Fisher Scientific, 12183025) and quality controlled with protocols from Illumina. A total amount of 2 μg RNA per sample was used as input material for library construction. Strand-specific sequencing libraries were generated through the dUTP method by using the resulting RNA from NEBNext Ultra Directional RNA Library Prep Kit for Illumina. RNA-Seq was performed on an Illumina HiSeq 2000 platform, and 100 bp paired-end reads were generated. In mRNA-Seq analysis, differential RNA expression patterns are identified through DESeq2 and EdgeR algorithms (42, 43). Gene Ontology (GO) and Kyoto Encyclopedia of Genes and Genomes (KEGG) analysis were conduct by WebGestalt (44).

### ChIP-Seq.

CCA cells were treated with different drugs (vehicle, BETp, mTORi, and BETp + mTORi) for 48 hours. After harvest, cells were crosslinked with 1% formaldehyde for 10 minutes at room temperature, and the reaction was quenched with 125 mM glycine. After chromatin were fragmented to 200–600 bp by sonication, chromatin templates from 10 million cells were used for ChIP experiments. Samples were immunoprecipitated with 2 μg of H3K27ac (Abcam, ab4729) antibody overnight at 4°C followed by washing steps. After reverse cross-linking, the ChIP DNA fragment was purified and repaired using End-It DNA End-Repair Kit, followed by treatment with Taq polymerase to generate single-base 3′ overhangs used for adaptor ligation. Following the ligation of a pair of Illumina adaptors to the repaired ends, the ChIP DNA were amplified using the adaptor primers. ENCODE guidelines were used to maintain the quality of all data sets, including the read depth (45). We identified genomic loci with peaks that were enriched for H3K27ac of every sample by *MACS* (46). A pipeline had been established in which *fastq* files were analyzed to output bed files containing enriched peaks. Through this pipeline, sequences underwent FastQC tool for quality-control measures and were aligned to the reference genome (hg19) using the *Bowtie2* tool (47), and MACS2 algorithms determined H3K27Ac peak enrichment. For global H3K27Ac enrichment analysis, BED files were generated using ± 50 bp of the enrichment summit, and plots were generated using seqMINER (48).

### PSAT1 shRNA knockdown.

The shRNAs for PSAT1 were purchased from MilliporeSigma in the pLKO.1-puro backbone. Primer sequences are as follows: shRNA-1 (5′–3′): GCACTCAGTGTTGTTAGAGAT; shRNA-2 (5′–3′): GCCAAGAAGTTTGGGACTATA; shRNA-3 (5′–3′): GCACTCAGTGTTGTTAGAGAT; and nontargeting control (5′–3′): CCGGCGCTGAGTACTTCGAAATGTCCTC. The shRNA plasmids were mixed with psPAX and VSVG for virus packaging in 293T cells. After the collection of media containing the virus, the virus was added to SNU1079 with polybrene and was then selected in puromycin.

### Quantitative analysis of one carbon metabolites.

Metabolite extracts were prepared and analyzed by LC coupled with high-resolution MS (LC-HRMS). SNU1079 cells were treated with different drugs (vehicle, BETp, mTORi, and BETp + mTORi) for 48 hours. After removing the media, plates were washed with cold PBS 3 times. A total of 10 mL liquid nitrogen was added in each plate to freeze cells. Ice-cold HEPES buffer (0.3 mL) was put in each plate to extract metabolites. After sonication in ice with 3 cycles of 30 seconds on and 30 seconds off, extracts were centrifuged at 17,000*g* for 5 minutes at 4°C, and supernatants were collected through a 10 kDa cut-off filter at 17,000*g* for 10 minutes at 4°C. In total, 10 μL of supernatant was injected into a Thermo Vanquish LC system containing a Phenomenex Synergy Hydro-RP 2.0 × 100 mm column with 2.5 μm particle size. Mobile phase A (MPA) was 0.1% formic acid in 10 mM ammonium formate. MPB was 5 mM ammonium formate in methanol. The flow rate was 200 μL/min (at 35°C), and the gradient conditions were: initial 5% MPB, increased to 90% MPB at 10 minutes, held at 90% MPB for 5 minutes, returned to initial conditions, and equilibrated for 5 minutes. The total run time was 20 minutes. Data were acquired using a Thermo Orbitrap Exploris 240 mass spectrometer under ESI^+^ ionization mode at a resolution of 240,000 with full-scan mode. Raw data files were imported into Thermo Trace Finder software for final analysis. The relative abundance of each compound was normalized by DNA concentrations.

### In vivo xenograft experiment.

Twenty NCr-Foxn1 nude (NCRNU) female mice (Taconic Biosciences) at 4–6 weeks were implanted with 5M SNU1079 in 100 μL HBSS s.c. at both left and right flanks. Drug administration began when 85% tumors size reached 100 mm^3^, which took 3 weeks. Mice were divided into 4 groups, including vehicle group (*n* = 8, 100 μL of 30% SBE-β-CD by s.c. administration with q5d2), BETp group (*n* = 8, 100 μL of 30 mg/kg ARV771 in 30% SBE-β-CD by s.c. administration with q5d2), mTORi group (*n* = 8, 100 μL of 20 mg/kg/day AZD8055 in 30% SBE-β-CD by oral gavages with q5d2), and BETp + mTORi group (*n* = 10, the combinational administration in BETp group and mTORi group). All tumors were measured every 4 days. One-way ANOVA was used to calculate the significance between different groups.

### IHC and TUNEL assay.

As previous described (49), FFPE slides baked at 60°C for 2 hours before being deparaffifinized and hydrated by dimethylbenzene (Sigma-Aldrich, 1330-20-7) and ethanol (Sigma-Aldrich, 64-17-5). For IHC, antigens were then repaired in citrate buffer (Sigma-Aldrich, C9999). Endogenous peroxidase was blocked with 3% H_2_O_2_, and then slides were blocked with bovine serum albumin (BSA) (A1595, MilliporeSigma). Next, the slides were incubated overnight along with the indicated primary antibodies. The next day, the slides were incubated together with the secondary antibodies. Samples were stained by dimethylbenzene (521116, Sigma-Aldrich) and hematoxylin (51275, Sigma-Aldrich); they were then sealed with neutral gum. TUNEL assay was conducted by following the TUNEL assay kit (MilliporeSigma, C10617). Slides were deparaffinized and permeabilized with 4% paraformaldehyde for 15 minutes at 37°C. TdT reaction and Click-iT Plus reaction were conducted according to the protocol. A fluorescence microscope with 495 nM excitation was used to detect TUNEL, and 350 nM was used to detect DAPI. Expression quantitation were conducted by ImageJ (version1.53; NIH).

### Statistics.

Prism 9 software was used to analyze and present quantitative data with the format of mean ± SD. *P* < 0.05 is considered significant. One-way ANOVA was used for analyzing data with 3 or more groups.

### Study approval.

All animal studies were approved by the IACUC at MD Anderson Cancer Center (protocol no. 00001469-RN02).

### Data availability.

Values for all data points in graphs are reported in the Supporting Data Values file. The RNA-Seq and ChIP-Seq data are available at GEO (GSE248632 and GSE248633).

## Author contributions

YZ contributed methodology, data analysis and visualization, manuscript writing, and editing; DZ contributed methodology, data analysis and visualization; SC contributed methodology and data analysis; YHJ contributed methodology; PBM contributed data analysis and visualization; MC contributed data analysis and visualization; SB contributed methodology; PS contributed methodology; JAC contributed methodology; LT contributed methodology; PLL contributed methodology; MJ contributed funding and conceptualization; JTH contributed conceptualization; JR contributed data analysis; TH contributed conceptualization and study design; LNK contributed conceptualization and study design, methodology, data analysis and visualization, manuscript writing, reviewing, and editing.

## Supplementary Material

Supplemental data

Supplemental tables 1-5

Supporting data values

## Version 1 12/07/2023

In-Press Preview

## Version 2 01/23/2024

Electronic publication
