# Supplementary material for: CRISPR screening identifies BET and mTOR inhibitor synergy in cholangiocarcinoma through serine glycine one carbon
Source: JCI Insight. 2024 Jan 23;9(2):e174220. doi: 10.1172/jci.insight.174220 (PMC10906219; doi:10.1172/jci.insight.174220)
Supplement: Supplemental data [file jciinsight-9-174220-s193.pdf]

S. Figure1

A

SNU1079 ARV825(0 nM) ARV825(40 nM)

RAPA (0 nM)

RAPA (10 nM)

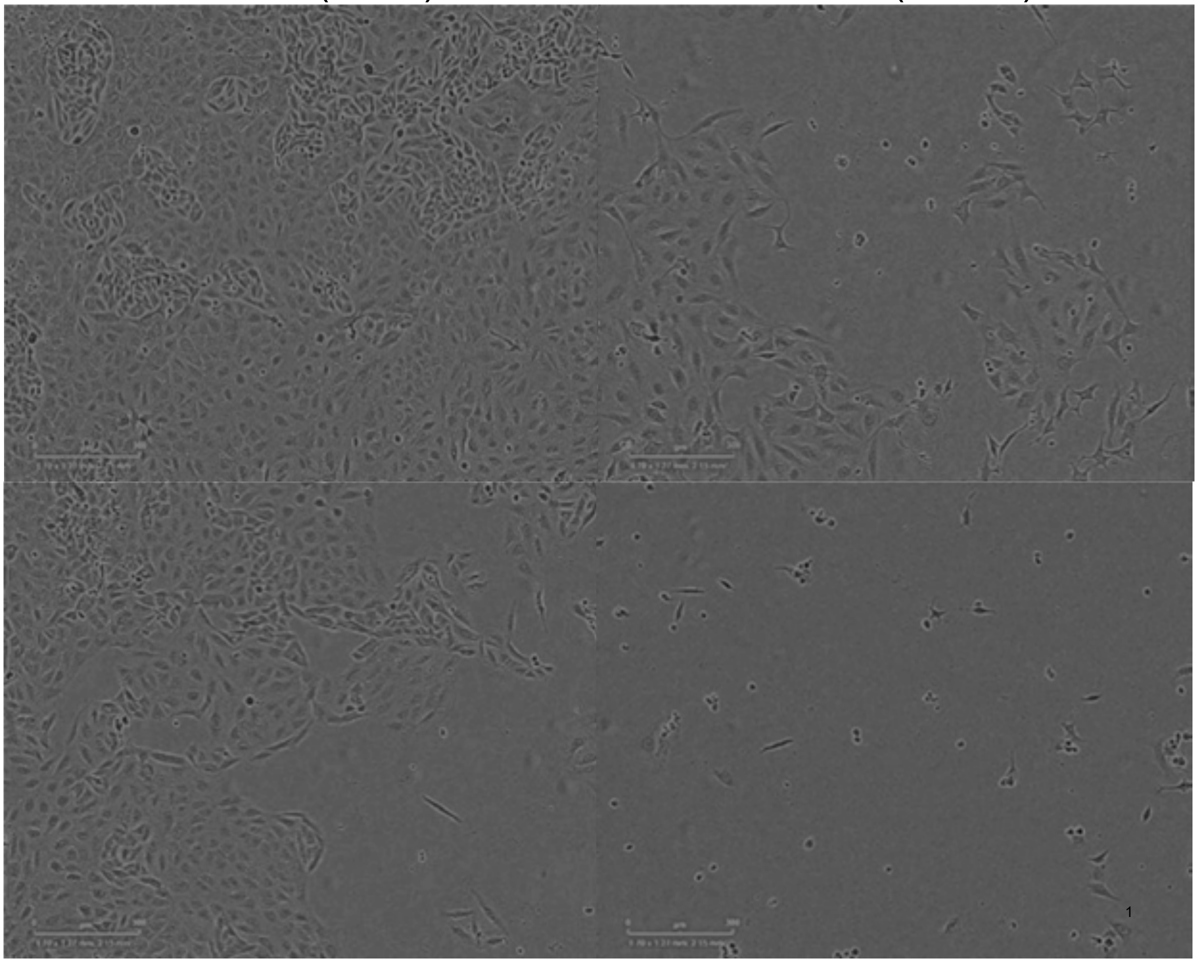

B

SNU1079 ARV825(0 nM) ARV825(40 nM)

AZD8055 (0 nM)

AZD8055 (50 nM)

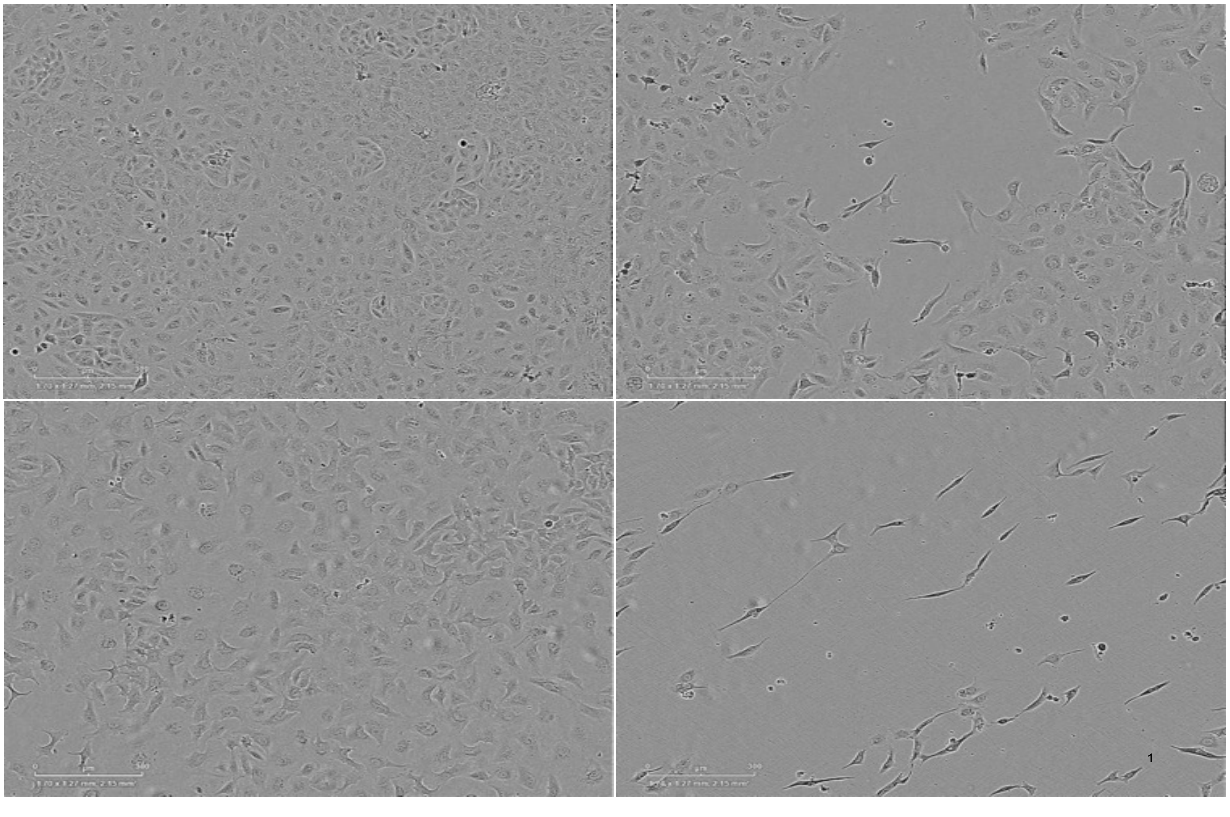

S Figure2

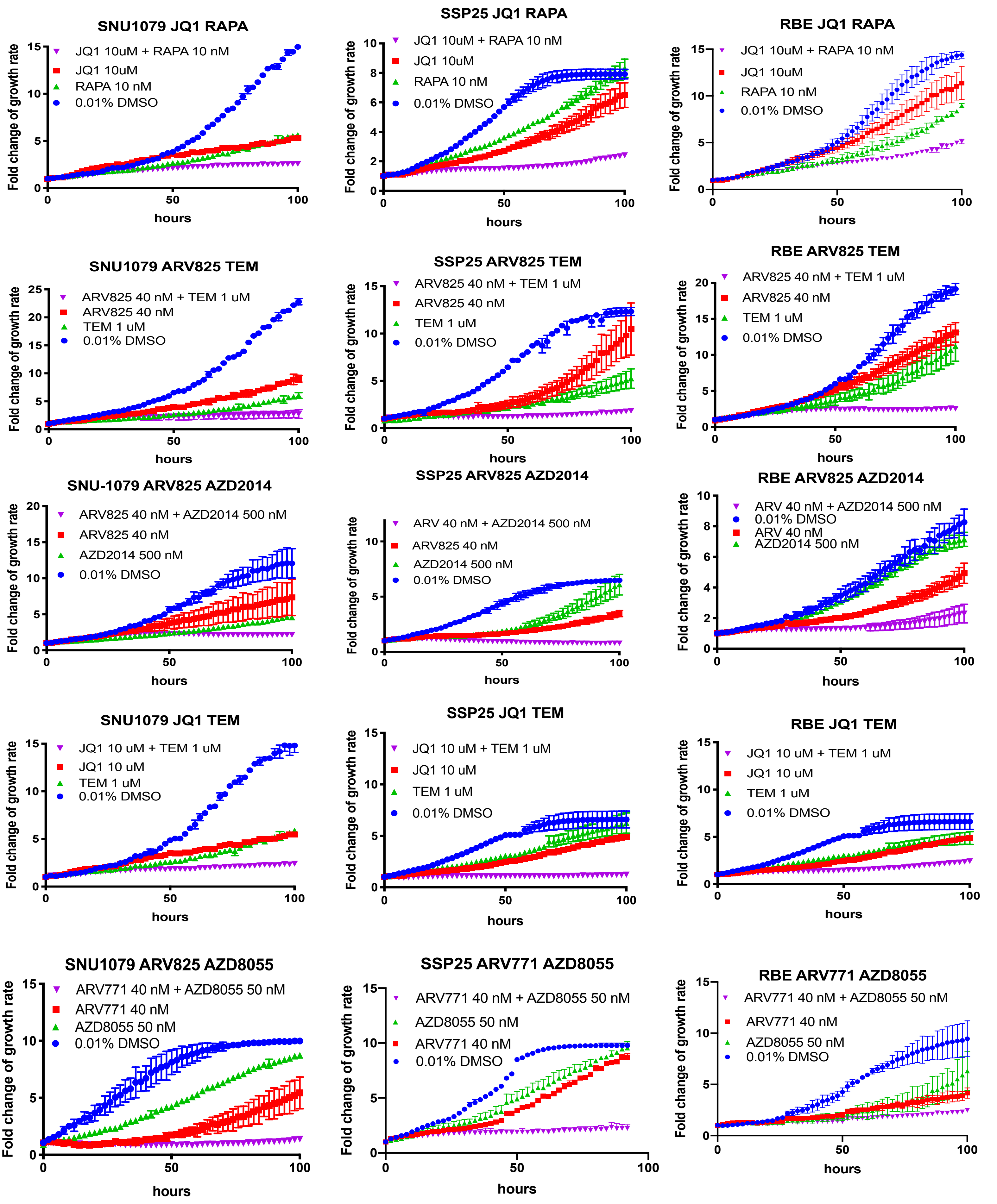
SNU1079 JQ1 RAPA
JQ1 10uM + RAPA 10 nM
JQ1 10uM
RAPA 10 nM
0.01% DMSO
SSP25 JQ1 RAPA
JQ1 10uM + RAPA 10 nM
JQ1 10uM
RAPA 10 nM
0.01% DMSO
RBE JQ1 RAPA
JQ1 10uM + RAPA 10 nM
JQ1 10uM
RAPA 10 nM
0.01% DMSO
SNU1079 ARV825 TEM
ARV825 40 nM + TEM 1 uM
ARV825 40 nM
TEM 1 uM
0.01% DMSO
SSP25 ARV825 TEM
ARV825 40 nM + TEM 1 uM
ARV825 40 nM
TEM 1 uM
0.01% DMSO
RBE ARV825 TEM
ARV825 40 nM + TEM 1 uM
ARV825 40 nM
TEM 1 uM
0.01% DMSO
SNU-1079 ARV825 AZD2014
ARV825 40 nM + AZD2014 500 nM
ARV825 40 nM
AZD2014 500 nM
0.01% DMSO
SSP25 ARV825 AZD2014
ARV 40 nM + AZD2014 500 nM
ARV825 40 nM
AZD2014 500 nM
0.01% DMSO
RBE ARV825 AZD2014
ARV 40 nM + AZD2014 500 nM
0.01% DMSO
ARV 40 nM
AZD2014 500 nM
SNU1079 JQ1 TEM
JQ1 10 uM + TEM 1 uM
JQ1 10 uM
TEM 1 uM
0.01% DMSO
SSP25 JQ1 TEM
JQ1 10 uM + TEM 1 uM
JQ1 10 uM
TEM 1 uM
0.01% DMSO
RBE JQ1 TEM
JQ1 10 uM + TEM 1 uM
JQ1 10 uM
TEM 1 uM
0.01% DMSO
SNU1079 ARV825 AZD8055
ARV771 40 nM + AZD8055 50 nM
ARV771 40 nM
AZD8055 50 nM
0.01% DMSO
SSP25 ARV771 AZD8055
ARV771 40 nM + AZD8055 50 nM
AZD8055 50 nM
ARV771 40 nM
0.01% DMSO
RBE ARV771 AZD8055
ARV771 40 nM + AZD8055 50 nM
ARV771 40 nM
AZD8055 50 nM
0.01% DMSO
Fold change of growth rate
hours

S Figure3

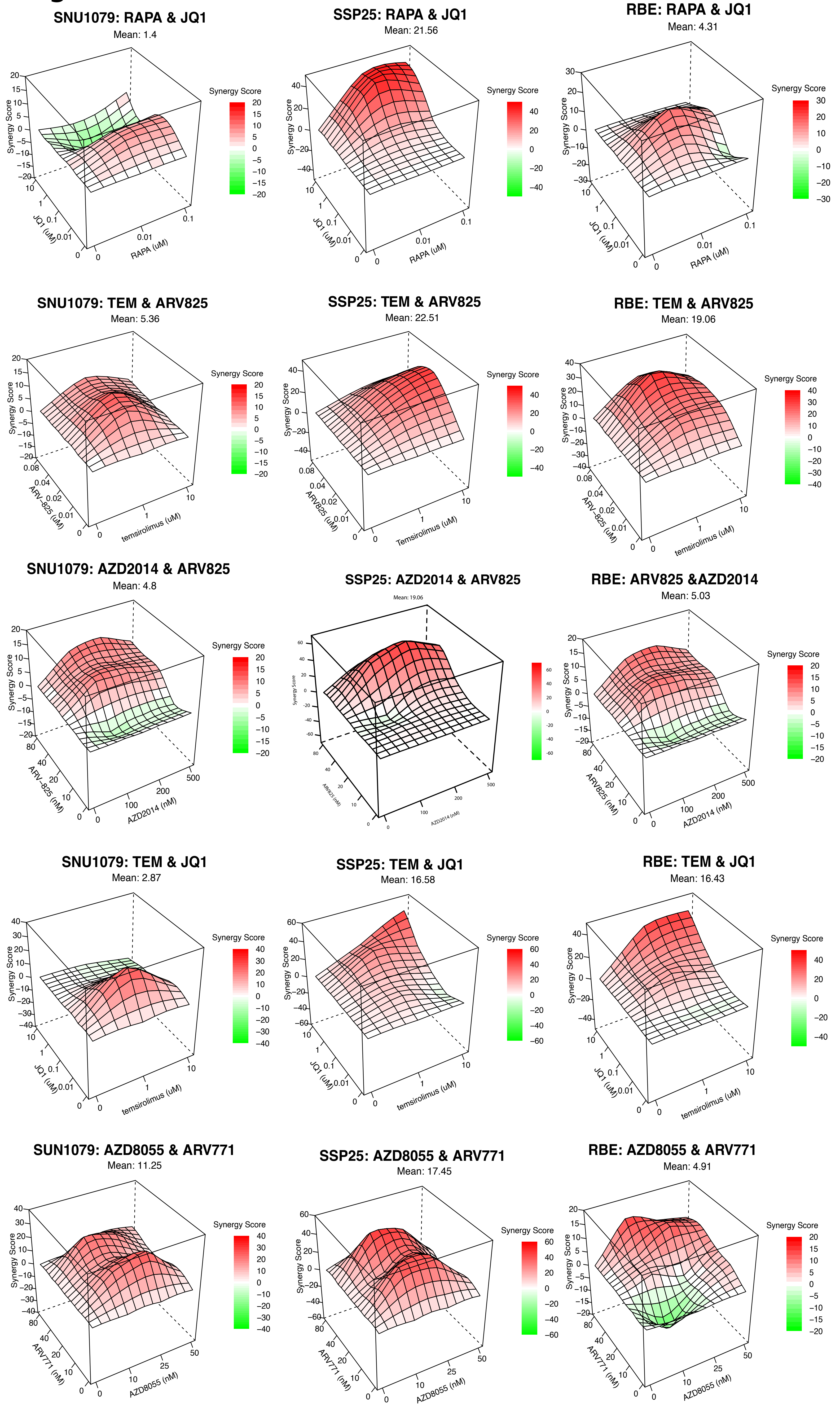

SNU1079: RAPA & JQ1
Mean: 1.4
Synergy Score
JQ1 (uM)
RAPA (uM)
SSP25: RAPA & JQ1
Mean: 21.56
Synergy Score
JQ1 (uM)
RAPA (uM)
RBE: RAPA & JQ1
Mean: 4.31
Synergy Score
JQ1 (uM)
RAPA (uM)
SNU1079: TEM & ARV825
Mean: 5.36
Synergy Score
ARV−825 (uM)
temsirolimus (uM)
SSP25: TEM & ARV825
Mean: 22.51
Synergy Score
ARV825 (uM)
Temsirolimus (uM)
RBE: TEM & ARV825
Mean: 19.06
Synergy Score
ARV−825 (uM)
temsirolimus (uM)
SNU1079: AZD2014 & ARV825
Mean: 4.8
Synergy Score
ARV−825 (nM)
AZD2014 (nM)
SSP25: AZD2014 & ARV825
Mean: 19.06
Synergy Score
ARV825 (nM)
AZD2014 (nM)
RBE: ARV825 &AZD2014
Mean: 5.03
Synergy Score
ARV825 (nM)
AZD2014 (nM)
SNU1079: TEM & JQ1
Mean: 2.87
Synergy Score
JQ1 (uM)
temsirolimus (uM)
SSP25: TEM & JQ1
Mean: 16.58
Synergy Score
JQ1 (uM)
temsirolimus (uM)
RBE: TEM & JQ1
Mean: 16.43
Synergy Score
JQ1 (uM)
temsirolimus (uM)
SUN1079: AZD8055 & ARV771
Mean: 11.25
Synergy Score
ARV771 (nM)
AZD8055 (nM)
SSP25: AZD8055 & ARV771
Mean: 17.45
Synergy Score
ARV771 (nM)
AZD8055 (nM)
RBE: AZD8055 & ARV771
Mean: 4.91
Synergy Score
ARV771 (nM)
AZD8055 (nM)

S Figure4

A

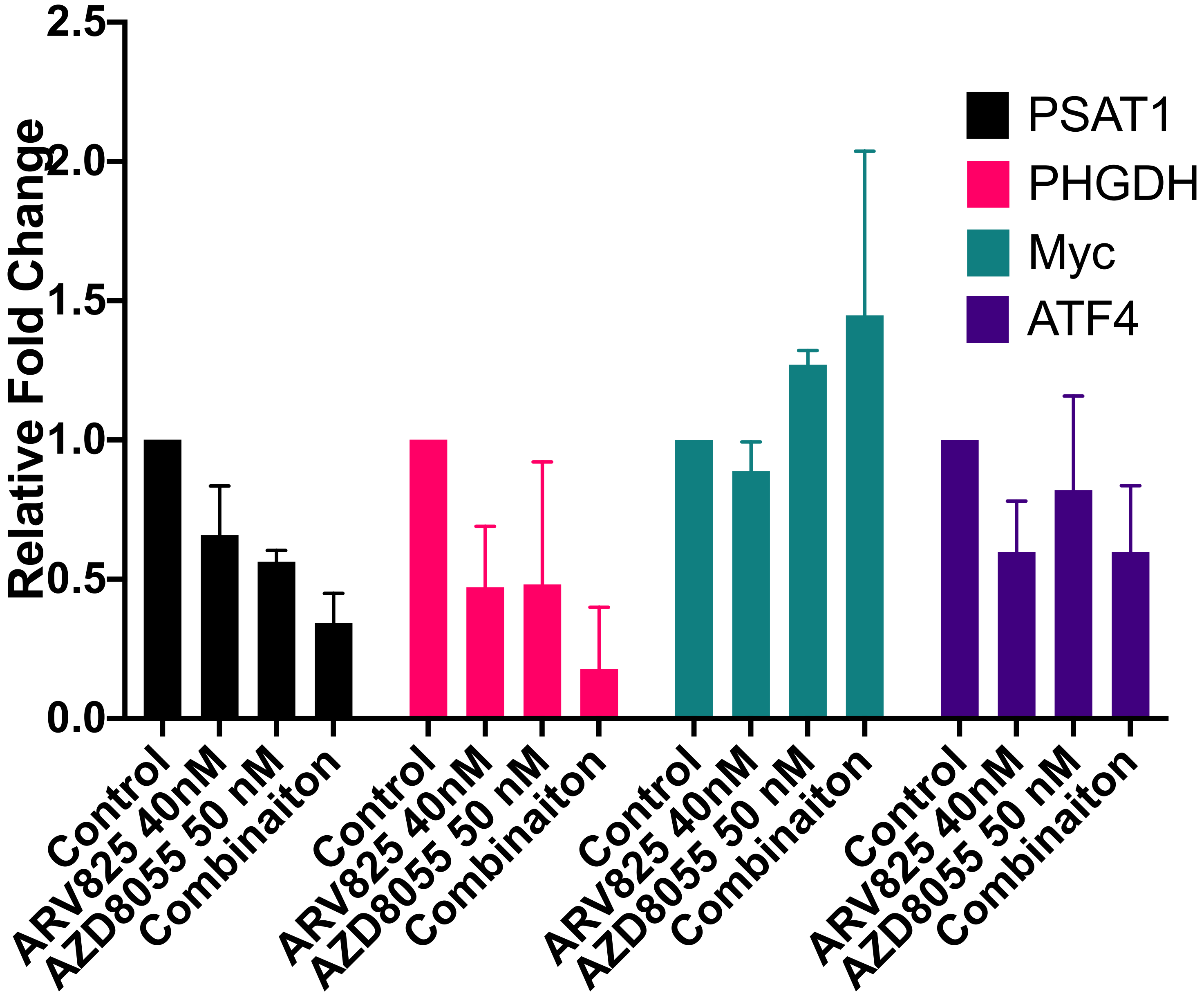

B

SNU1079

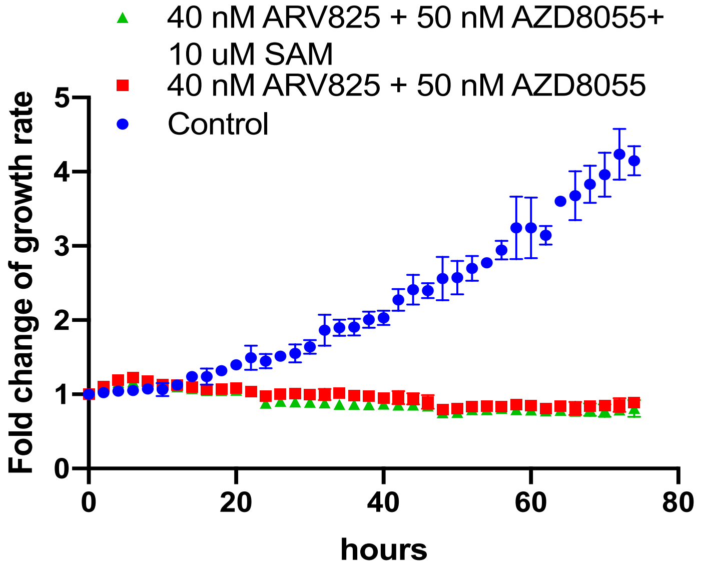

SSP25

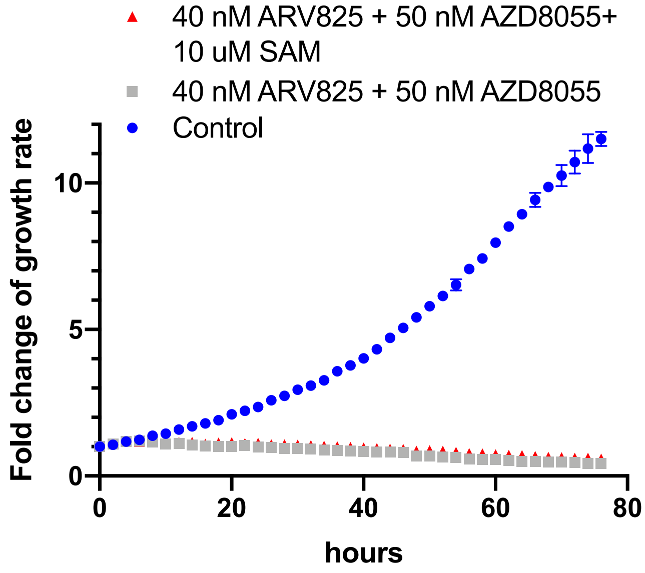

C

SNU1079

40 nM ARV825, 50 nM AZD8055,

0 uM SAM

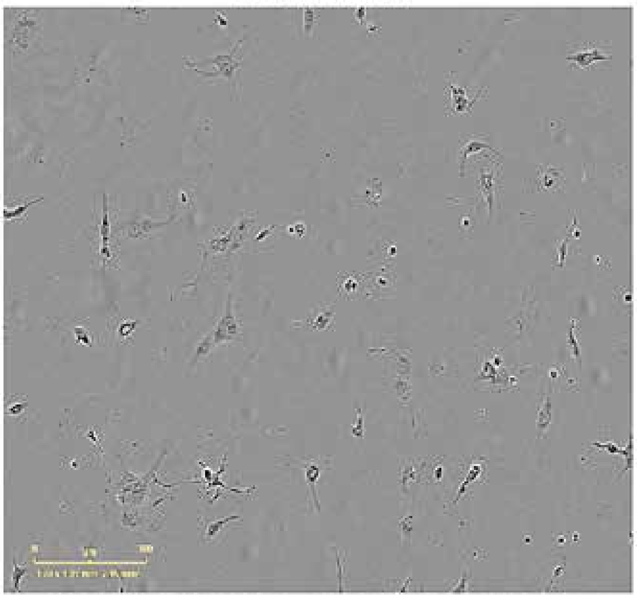

SNU1079

40 nM ARV825, 50 nM AZD8055,

100 uM SAM

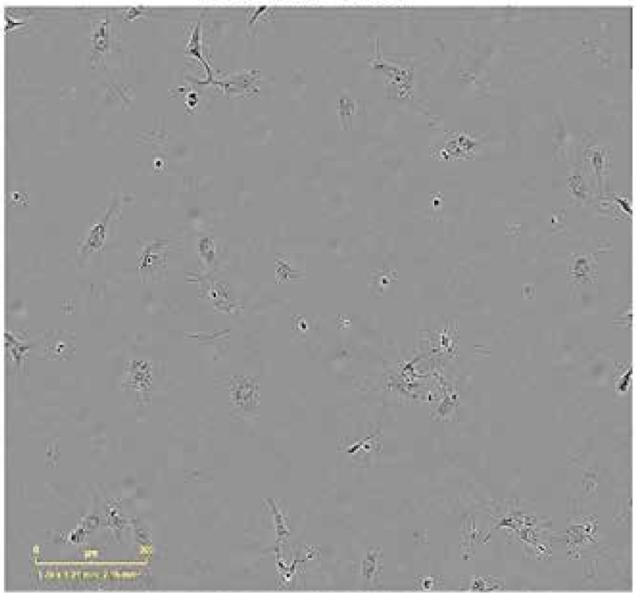

S Figure5

A

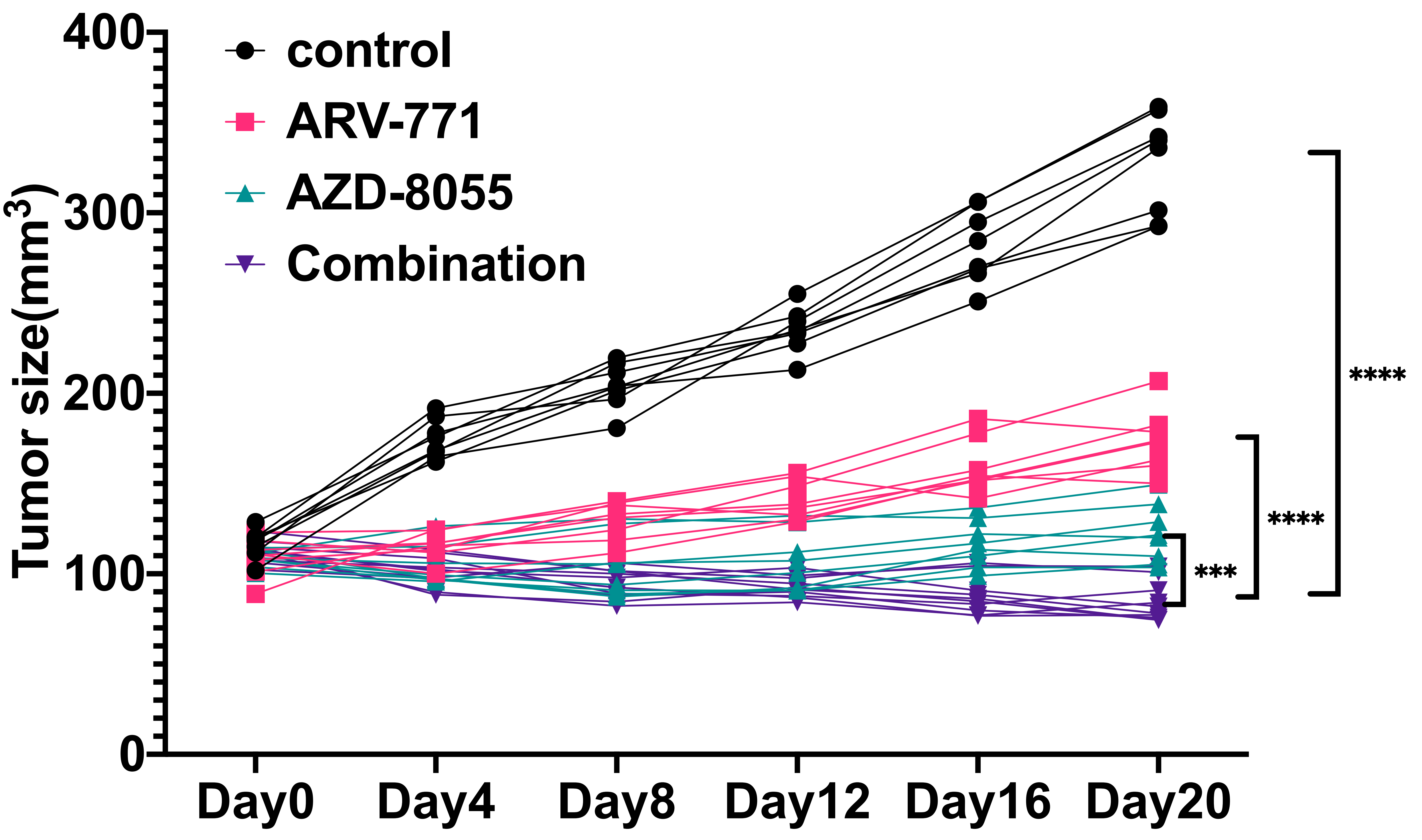

control
ARV-771
AZD-8055
Combination
Tumor size(mm^3)
400
300
200
100
0
Day0
Day4
Day8
Day12
Day16
Day20
****
****
***

B

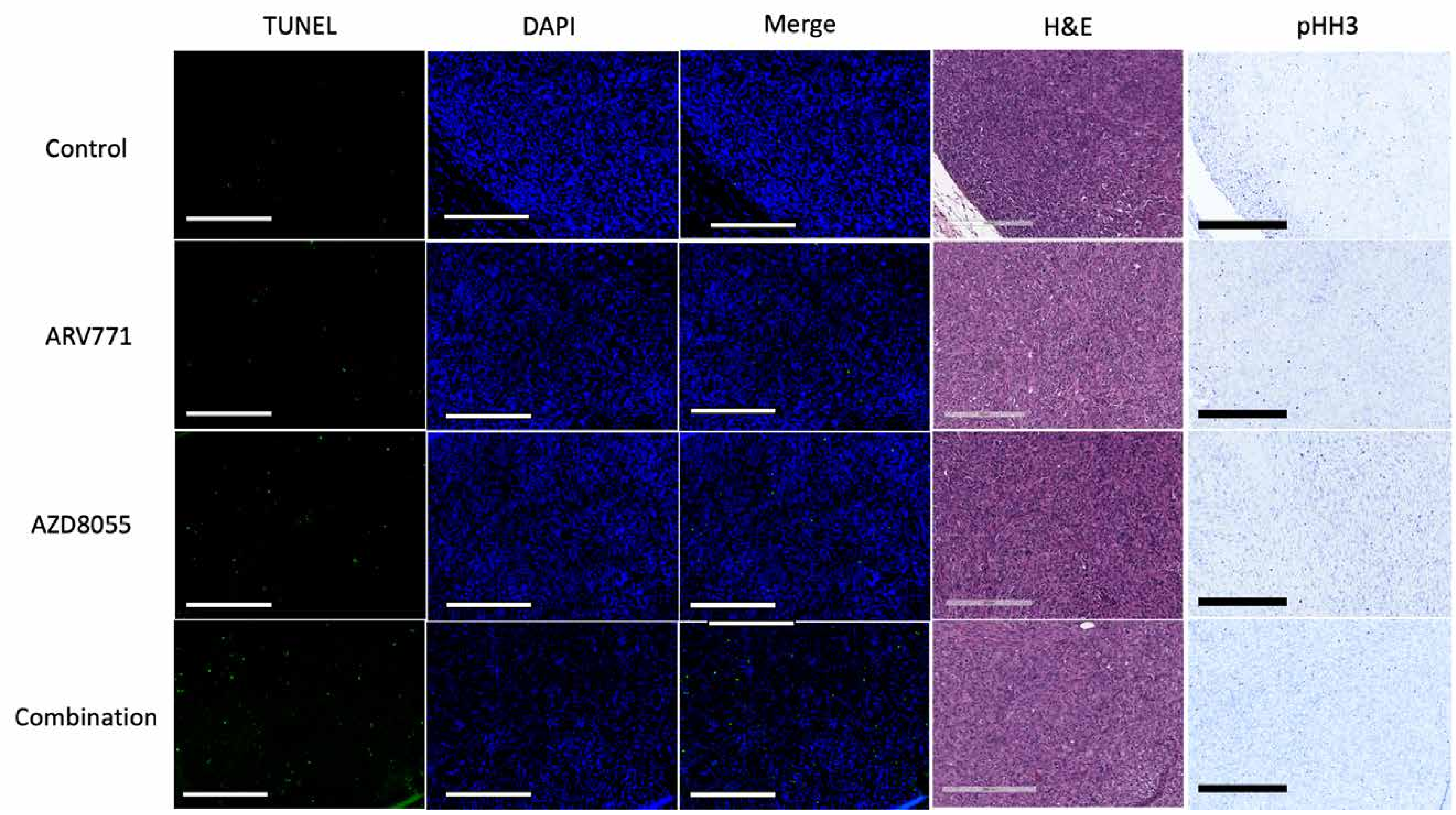

TUNEL
DAPI
Merge
H&E
pHH3
Control
ARV771
AZD8055
Combination
